
JOURNAL INFORMATION
==============================
NLM Title Abbreviation: Inter Econ
Journal ID: Inter Econ
NLM Journal ID: 101086399

Inter Economics

ISSN: 0020-5346

ARTICLE INFORMATION
==============================
PMCID: PMC7584870
DOI: 10.1007/s10272-020-0916-y
Article ID: 916
Article version: 1
Subjects: Forum

Lessons From the COVID-19 Crisis for Euro Area Fiscal Rules

Gros Daniel danielg@ceps.be

grid.22793.3d 0000 0004 0609 4239 Centre for European Policy Studies (CEPS), 1 Place du Congrès, 1000 Brussels, Belgium
Electronic publication date: 2020 Oct 24
Print publication date: 2020
Volume: 55
Issue: 5
First page: 281
Last page: 284
Copyright: © The Author(s) 2020
License: Open Access: This article is distributed under the terms of the Creative Commons Attribution 4.0 International License (https://creativecommons.org/licenses/by/4.0/).
Open Access funding provided by ZBW — Leibniz Information Centre for Economics.
License URL: https://creativecommons.org/licenses/by/4.0/

issue-copyright-statement: © The Author(s) 2020

==============================

The main thrust of the existing rules, although they were not really enforced, did go in the right direction, namely they were intended to reduce deficits and debts. The countries that stick to these principles went into the coronavirus crisis with lower debt and more fiscal space. Germany's fixation with the *schwarze Null*, or black zero, must now appear in a different light. It was always meant to be an appropriate stance for an economy close to full employment. By sticking to it during good times, the German government has been in a very strong position to react forcefully to this unprecedented crisis.

The real issue now is not whether the old rules should be reinstated immediately. Nobody argues that in 2021 the government should keep deficits below 3% of GDP. But some principles are still needed – unless one assumes that debt is free. Few would argue that governments can spend as much as they want. Even proponents of a permanent stimulus seem to recognise that fiscal space has its limits. Looking at the US for example, Krugman (2020) argues for a permanent stimulus in the form of a permanent increase in investment spending, broadly defined, of 2% of GDP without increasing taxes. He acknowledges that this would push the US debt-to-GDP ratio towards 200%, which he considers sustainable at today's low interest rates. Even this proposition contains some implicit consideration about sustainable debt levels. Why would a debt ratio of 300% be unacceptable? Why not go for deficit-financed investment spending of 3%-5% of GDP?

Some guidelines for fiscal policy are thus unavoidable. The key issue is what general considerations should guide fiscal policy in these uncertain times. I propose two:

- Large shocks might be more prevalent than we thought before the crisis. This would reinforce the case for 'keeping some powder dry', i.e. limiting the increase in debt during the crisis, and reducing debt levels when normality is restored.
- This time is different. The overall output gap should not be used to guide aggregate fiscal policy as this crisis directly affects very specific sectors. Some output gap is unavoidable as long as the virus remains a threat. No amount of demand management can change this.

## Keep debt low in an uncertain world

Many take the current level of interest rates as a given and observe that it is lower than growth rates. Krugman (2020) constitutes just one particular example of this tendency. However, this neglects two risks. First of all, it is possible that future, post-pandemic, growth rates will be lower than those experienced over the last few years. This is not desirable, but it cannot be excluded. Second, one cannot rule out that inflation will also remain even more subdued than over the last few years, leading to much lower growth rates in nominal GDP. This happened in Japan, where nominal GDP barely grew over decades. One argument for large fiscal deficits is that even unconventional monetary policy is no longer effective. But if monetary policy has indeed become ineffective in increasing inflation, one cannot exclude that in a post-pandemic environment, nominal GDP growth rates might be much lower than in the past, making it more difficult to reconcile high deficits and stable debt levels.

At the same time, this crisis has shown that a low level of debt (and deficit) provides a much stronger base for governments to respond to large shocks. The coronavirus crisis is already the second 'once-in-a-century' crisis to hit the euro area in the space of one decade. One certainty of this crisis is that nobody can know for sure about the distribution of large shocks and how likely it is that another shock materialises any time soon. But, as Kozlowski et al. (2020) argue, having experienced two pandemics in the space of 140 years must lead one to re-evaluate the likelihood of another one occurring during the next generation.

This does not mean that fiscal policy today should prepare for the next pandemic. The next major shock is likely to be quite different. But this heightened uncertainty suggests that prudent policy should keep debt levels well below the level that might be sustainable if the current constellation of ultra-low interest rates and moderate growth rates were to continue forever.

In this tug of war between low interest rates and increased uncertainty, the latter is more important. Interest rates have fallen only marginally, whereas the uncertainty about the outlook for future nominal growth rates has increased and the likelihood of large future shocks has risen dramatically. Letting the debt ratio explode towards 200% would be reckless.

## The sectoral specificity of the COVID-19 recession[1]

As argued above, the COVID-19 crisis is different from 'normal' recessions. Even if the first and second waves can be contained, some restrictions on mobility and hesitancy of consumers to buy some services requiring close contact will remain. This implies that some sectors of the economy will remain weak for some time, while others bounce back.

### Model-based analysis of the policy implications of a sectoral recession

Two recent papers analyse the sectoral nature of the COVID-19 recession in a formal model. One, Guerrieri et al. (2020) considers the pandemic crisis as a supply shock to a specific sector. The authors' main insight is that "a 50% shock that hits all sectors is not the same as a 100% shock that hits half the economy".

The conclusion associated with the sectoral nature of the shock has several implications for policy. One is that standard aggregate fiscal stimulus becomes less effective than usual because the sectoral shutdown mutes the Keynesian multiplier feedback. This is straightforward. An increased purchasing power might result in more spending on the sectors not affected by COVID-19, such as durable goods. This might result in excess demand for these goods. But the output gap will persist as long as service sectors requiring close contact remain closed.

1 This section is based in large part on Gros (2020).

Another recent contribution argues that COVID-19 should not be considered only as a sectoral supply shock (because of government-ordered social distancing measures), but also as a sectoral demand shock as households and firms voluntarily reduce demand for travel, tourism and other contact-intensive services. Farhi and Baqaee (2020) study supply and demand shocks in a general disaggregated model across multiple sectors. A major element in their approach is the input-output linkages across sectors that propagate these sectoral shocks (both demand and supply) to the entire economy.

Their major finding is that "aggregate demand stimulus is only about a third as effective as in a typical recession". This finding applies to both fiscal and monetary policy. The authors also argue, "More targeted forms of demand stimulus deliver better bang for the buck".

### Fiscal policy should not target the output gap in a sectoral recession

In the current situation, governments need to look forward to a longer period during which they need to provide replacement income for those rendered idle by the direct and indirect impact of the pandemic. Moreover, some GDP gap will persist since a number of important sectors will operate below normal capacity for some time. The question for macroeconomic policy is thus whether government should go beyond providing replacement income and try to lift aggregate demand.

Common sense suggests that no amount of support to aggregate demand can bring the sectors affected by COVID-19 back to their previous level. Those previously employed in travel would remain unemployed, even if spending on durable goods were to increase greatly.

Moreover, economic modelling suggests that consumers might be more careful in spending today when there are some goods that they temporarily cannot or do not want to buy. Most economic models suppose that many households are cash constrained and will spend a good portion of any transfer they receive from the government. However, this mechanism does not work as well when households today cannot afford their normal consumption basket.

An example can illustrate this proposition.[2] Consider a person who wants to buy new sports equipment or clothes to use in a gym or on holiday abroad. Normally, a higher income would make it more likely that the entire consumption basket (vacation and sports equipment) is bought today. But if today, due to the pandemic, foreign travel is impossible and gyms are closed, the sports equipment and clothes will not be bought. Higher cash transfers would probably just lead to more savings. The counterpart to higher government deficits (over and above those needed to provide substitute incomes) would thus lead mainly to higher savings – as it occurred this summer.

The observation that aggregate demand stimulus becomes less efficient in a sectoral downturn implies that one should not judge deficits by the metrics used during normal recessions; namely by relating the deficit to the output gap or the unemployment rate. Both metrics are misleading in the current circumstances. Any remaining fiscal space should be used to support adjustment and new jobs, rather than to try to fill the bank accounts of households with transfers they are likely to save. Coibon et al. (2020) find that US consumers saved most of the transfers distributed under the US Coronavirus Aid, Relief, and Economic Security (CARES) Act.

With much lower multipliers, it also becomes more difficult to argue that austerity would be 'self-defeating' because higher deficits increase demand and GDP so much that the denominator in the debt-to-GDP ratio falls. This argument does not hold in the longer-term perspective as shown in Gros (2011), but it becomes even more shaky when multipliers are low to begin with.

Aggregate fiscal policy multipliers are likely to remain low for some time. But it is possible, indeed even likely, that they will increase again once the sectoral health concerns have been overcome. Fiscal multipliers are thus likely to be higher tomorrow than today. Blanchard and Leigh (2013) argue convincingly:

> Large multipliers do not necessarily affect the optimal timing of fiscal consolidation .... If they remain just as large in the future, the adjustment will be as painful later. But, if they are larger now than later, this tilts the adjustment toward doing more later: Less pain now, less pain later.

The logic of this argument is that it is the time path of the multiplier that should inform the consolidation effort. Blanchard and Leigh (2013) wrote at a time when one could make the case that multipliers were high given financial market dislocations and the then widespread expectation was that interest rates would 'normalise' soon. What is different today is that multipliers are likely to be lower today than tomorrow. The logic of this argument would thus imply more fiscal consolidation today and less tomorrow.

2 The extreme example made by Keynes is that of shoes: If only right-foot shoes are available today, consumers will not buy them; they would rather wait until both right and left shoes are available again as pairs. For this reason, interest rates will not have a big impact on consumption decisions.

## Conclusions

It is easy to shoot down the idea that the pre-COVID-19 fiscal rules could be somehow resurrected without any change. This seems impossible also for purely technical reasons. For example, one key element of the improved Stability Pact has been the emphasis on cyclically adjusted deficits.

The difficulties with all calculations of potential output and its growth are obviously magnified by the coronavirus crisis. A mechanical implementation of the econometric procedures used so far to recover potential output from past data about GDP would lead to a sharp drop in the level of potential output, including presumably revisions in past output gaps.

A mechanical resurrection of the old rules is thus out of the question. But the fundamental question remains: Should governments try to limit the increase in the unavoidable run-up in debts, or should they just run large deficits for as long as interest rates remain low?

Limiting the deficits and increases in the debt level does not imply that governments should immediately cut expenditure. The prudent course of action would be to limit expenditure to the provision of replacement income for workers as long as needed, but not to try to increase deficits beyond this level in the vain hope of having a substantial impact on the recovery in the short run. If aggregate private demand remains weak even after the health crisis has been overcome, one could make the case for some continuing deficits. Whether or not this will be the case in a few years is impossible to predict today. What is certain, however, is that those governments who have not used up all of their fiscal space today will be better prepared for that eventuality.

Daniel Gros, Centre for European Policy Studies, Brussels, Belgium.


References

Blanchard, O. and D. Leigh (2013, 3 May), Fiscal consolidation: At what speed?, VoxEU, https://voxeu.org/article/fiscal-consolidation-what-speed (16 September 2020).
Gros, D. (2011, 29 November), Can austerity be self-defeating?, VoxEU, https://voxeu.org/article/can-austerity-be-self-defeating (16 September 2020).
Gros, D. (2020), Benefits and drawbacks of an “expenditure rule, as well as of a “golden rule”, in the EU fiscal framework, Study for the European Parliament, https://www.europarl.europa.eu/RegData/etudes/STUD/2020/614523/IPOL_STU(2020)614523_EN.pdf (10 September 2020).
Gros, D. (2020), Europe and the Covid-19 crisis: the challenges ahead, CEPS Policy Insights, 2020–20, https://www.ceps.eu/ceps-publications/europe-and-the-covid-19-crisis/ (10 September 2020).
Krugman, P. (2020, 10 May), The case for permanent stimulus, VoxEU, https://voxeu.org/article/case-permanent-stimulus (9 September 2020).
Olivier C., Y. Gorodnichenko and M. Weber (2020, 8 September), How US consumers use their stimulus payment, VoxEU, https://voxeu.org/article/how-us-consumers-use-their-stimulus-payments (10 September 2020).
Farhi, E. and D. R. Baqaee (2020), Supply and Demand in Disaggregated Keynesian Economies with an Application to the Covid-19 Crisis, Mimeo.
Guerrieri, V., G. Lorenzoni, L. Straub and I. Werning (2020), Macroeconomic Implications of COVID-19: Can Negative Supply Shocks Cause Demand Shortages?, National Bureau of Economic Research Working Paper, w26918.
Kozlowski, J., L. Veldkamp and V. Venkateswaran (2020), Scarring Body and Mind: The Long-Term Belief-Scarring: Effects of COVID-19, Federal Reserve Bank of St. Louis, https://research.stlouisfed.org/wp/more/2020-009 (16 September 2020).
